# QuickStats

**Published:** 2013-12-13

**Authors:** Eunice Park-Lee, Vincent Rome, Christine Caffrey, Lauren Harris-Kojetin

**Figure:**
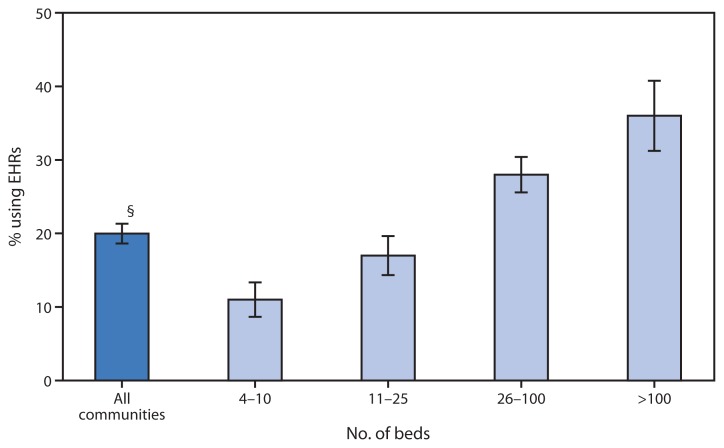
Percentage of Residential Care Communities^*^ Using Electronic Health Records (EHRs),^†^ by Number of Beds — National Study of Long-Term Care Providers, United States, 2012 ^*^ Assisted living and similar communities (e.g., personal care homes, adult care homes, board and care homes, and adult foster care). Residential care communities with missing data were excluded. ^†^Participating administrators and directors were asked, “An electronic health record is a computerized version of the resident’s health and personal information used in the management of the resident’s health care. Other than for accounting or billing purposes, does this residential care community use electronic health records?” ^§^95% confidence interval.

In 2012, 20% of residential care communities used EHRs. Greater proportions of communities with larger numbers of beds used EHRs compared with communities with fewer beds. Communities with >100 beds (36%) were more than three times as likely as communities with 4–10 beds (11%) to use EHRs.

**Source:** National Study of Long-Term Care Providers, 2012. Available at http://www.cdc.gov/nchs/nsltcp.htm.

